# Polyarthritis and Psoriasiform Skin Lesions following Pembrolizumab Therapy

**DOI:** 10.31138/mjr.32.4.367

**Published:** 2021-12-27

**Authors:** Vasiliki Koulouri, Michalis V. Karamouzis, Clio P. Mavragani

**Affiliations:** 1 Department of Physiology, National and Kapodistrian University of Athens, School of Medicine, Athens, Greece,; 2 Molecular Oncology Unit, Department of Biological Chemistry, National and Kapodistrian University of Athens, School of Medicine, Athens, Greece,; 3 Evgenidio Treatment Center, “Agia Trias”, National and Kapodistrian University of Athens, School of Medicine, Athens, Greece

**Keywords:** Immune checkpoint inhibitors, skin lesions, arthritis

## Abstract

A notable feature of immune checkpoint inhibitor (ICI) therapy in oncology patients is its association with increased frequency of immune related adverse reactions, directly associated with their unique mechanism of action. These adverse events are of great interest to rheumatologists, as not only do they commonly require immunosuppressive therapeutic intervention, but can also aid in unveiling important immunopathogenetic pathways that underlie autoimmune phenomena. Herein we describe a case of psoriasiform skin lesions and polyarthritis in a patient receiving ICI for lung cancer.

## INTRODUCTION

The use of immune checkpoint inhibitors (ICI) has brought upon a revolution in the treatment of certain oncology patients. Immune related adverse events are a rather common feature of ICI therapy. Our knowledge regarding the spectrum of ICI related autoimmune reactions is constantly expanding, with a growing body of evidence associating their manifestation with a favourable prognosis.

## CASE DESCRIPTION

**Figure 1.**
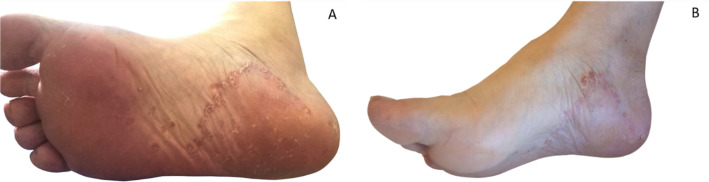
Psoriasiform skin lesions in a patient following pembrolizumab infusions. (A) At presentation; (B) After treatment with small doses steroids, at one month follow-up.

A 59-year-old man with a recent diagnosis of metastatic lung adenocarcinoma was referred for rheumatologic evaluation due to small-joint swelling and tenderness of both hands and feet of two-month duration, leading to significant pain and stiffness. Clinical examination revealed the presence of a non-pruritic rash with desquamative papules and plaques on an erythematous base, solely distributed on the soles of both feet and extending to the periungual area of several toes (**Figure 1A**). Prior to the current presentation, 3 infusions of pembrolizumab 200mg single-agent every 3 weeks had been administered since the programmed cell death ligand 1 (PDL-1) expression in tumour tissue was found to be 80%. No history of psoriasis, inflammatory bowel disease or recent infections was reported. Prednisolone at a dose of 10mg qd was prescribed with remarkable improvement in joint complaints and rash (**Figure 1B**) at one-month follow-up (**Figure 1B**). The patient is still being treated with single-agent pembrolizumab and his disease is radiologically in complete response.

## DISCUSSION

Pembrolizumab is an immune checkpoint inhibitor (ICI) targeted against programmed cell death protein 1 (PD-1) receptor on lymphocytes and along with other ICIs has been associated with frequent autoimmune adverse events. Autoimmune manifestations following ICI treatment have been suggested to serve as an indicator of favourable response in certain malignancies^2,3^, probably indicating effective immune system stimulation. Of interest, PD-1 expression on T cells was found to be higher in patients with psoriasis compared to healthy controls; however, among patients, PD-1 levels were inversely correlated with disease activity^4^ possibly reflecting an attempt to control immune hyper-reactivity. Additional studies in immune checkpoints may reveal important autoimmune pathogenetic pathways and enhance our knowledge regarding autoimmune pathogenesis.
